# Utilization of amplicon-based targeted sequencing panel for the massively parallel sequencing of sporadic hearing impairment patients from Saudi Arabia

**DOI:** 10.1186/s12881-016-0329-8

**Published:** 2016-10-10

**Authors:** Ashraf Dallol, Kamal Daghistani, Aisha Elaimi, Wissam A. Al-Wazani, Afaf Bamanie, Malek Safiah, Samira Sagaty, Layla Taha, Rawabi Zahed, Osama Bajouh, Adeel Gulzar Chaudhary, Mamdooh Abdullah Gari, Rola Turki, Mohammed Hussein Al-Qahtani, Adel Mohammed Abuzenadah

**Affiliations:** 1 Center of Innovations in Personalized Medicine, King Abdulaziz University, P.O. Box 80216, Jeddah, 21589 Kingdom of Saudi Arabia; 2 Center of Excellence in Genomic Medicine Research, King Abdulaziz University, Jeddah, Kingdom of Saudi Arabia; 3 King Abdulaziz University Hospital, King Abdulaziz University, Jeddah, Kingdom of Saudi Arabia; 4 Jeddah Institute for Speech and Hearing, Jeddah, Kingdom of Saudi Arabia; 5 King Fahad General Hospital, Jeddah, Kingdom of Saudi Arabia; 6 Faculty of Applied Medical Sciences, King Abdulaziz University, Jeddah, Kingdom of Saudi Arabia

**Keywords:** Hearing impairment, NGS, Targeted sequencing, Saudi Arabia

## Abstract

**Background:**

Hearing Impairment (HI) can have genetic or environmental causes and in some cases, an interplay of both. Genetic causes are difficult to determine as mutations in more than 90 genes have been shown recently to be responsible for HI. Providing a genetic diagnostic test for HI is therefore a challenge especially for ethnic groups where GJB2 mutations are shown to be rare.

**Results:**

Here we show the design and implementation of an amplicon-based targeted sequencing panel that allows the simultaneous sequencing of 87 HI genes. Mutations identified included known pathogenic mutations and novel variants with unknown significance. The diagnostic rate of this panel is 28 % when only pathogenic variants were reported. However, an additional 28 % harbored recurrent combinations of novel or rare single nucleotide variants in the OTOF or PCDH15 genes. Such combinations were not identified in healthy individuals.

**Conclusions:**

Targeted sequencing approach is a very useful strategy for the identification of mutations affecting the HI genes because of its relatively fast turn-around time and cost effectiveness compared to whole-exome sequencing. Further novel or rare variants could be identified by implementing a large-scale screening of HI using our panel which will eventual lead to a higher diagnostic rate.

## Background

Hearing loss is a common disease found in many populations worldwide ast is estimated that at least 50 % of prelingual hearing impairment (HI) is heritable either as a phenotype in many syndromes or in a non-syndromic fashion [1]. The heritability of HI follows all types of Mendelian inheritance and can be passed down to subsequent generations in autosomal/X-linked recessive or dominant manner. The large number of genes required for hearing causes such complexity in inheritance patterns. Mutations in any one of such genes is sufficient to cause various degrees of HI. Mutations in GJB2, SLC26A4, MYO15A, OTOF, and CDH23 genes are the most frequent causes of autosomal recessive HI at varying degrees [2]. On average, about 50 % of any given cohort of autosomal recessive HI will have a pathogenic mutation in the GJB2 gene [3–6]. However, the frequency of HI-causing GJB2 gene mutations is population-dependent as it is most predominant HI gene in Caucasians and rather rare in South East Asians or Arab populations [7, 8].

The prevalence of the 35delG mutation in GJB2 gene in Caucasians has allowed its use as a genetic screening tool for early detection and diagnosis of HI in newborns. However, such a test is not applicable in other ethnic groups due to its rarity. In fact, since over 90 genes have now been shown to be associated with HI, it is not feasible to offer classical genetic testing for HI in many populations. The advent of massively parallel sequencing and whole-exome sequencing (WES) led to a rapid increase in the rate of identifying the genetic determinants of HI [9]. In order to achieve such aim, whole-exome sequencing is applied to several members of the affected family in order to identify the genetic cause (s) of HI amongst hundreds of candidate variants [10, 11]. Furthermore, WES is expensive and time-consuming and it is not yet amenable for large-scale screening of HI that will invariably include sporadic cases. Amplicon-based targeted sequencing is a cost-effective tool to precede WES that will allow the focused sequencing of relevant HI genes and improve the diagnostic rate of such tests [12, 13].

In this study, we designed and validated a custom-made amplicon-based targeted sequencing panel for HI that allows the simultaneous sequencing of 87 genes shown previously to be associated with various forms of HI.

## Materials and methods

### Patients

Samples from 25 HI patients were collected following the appropriate local ethical protocols and guidelines from the Audiology clinic at King Abdulziz University Hospital in Jeddah, Saudi Arabia or the Audiology department of the King Fahad General Hospital, Jeddah Institute for Speech and Hearing (JISH) as well as Al-Amal secondary school for the hearing impaired, Jeddah, Saudi Arabia. All samples were selected according to the diagnosis of bilateral severe-to-profound hearing impairment as determined by audiologists in the study team. The patients ages ranged from 4 to 22 years old. Control samples were obtained from non-HI patients and aged 18–40 years old. DNA was extracted from peripheral blood DNA using the standard protocol of the QIAGEN Blood DNA Extraction kit. The study was approved by the local ethical committee.

### Targeted sequencing of deafness genes

Eighty-seven genes involved in HI were determined using the Deafness Variation Database (http://deafnessvariationdatabase.org). The gene names were submitted for primer design to the http://Ampliseq.com website were a targeted sequencing panel was designed and manufactured by Life Technologies. Barcoded Ampliseq libraries (2 pools) were prepared using the Ampliseq Library kit 2.0 from 10 ng of DNA (concentration determined using the Qubit™ fluorometer). Templated spheres were prepared using 100 pM of DNA from each library using the Ion OneTouch 2.0 machine. Template-positive spheres from the barcoded libraries were multiplexed and loaded onto Ion chips 316 or 318 version 2.0 and sequencing was performed using the Ion Sequencing 200 v2 kit from Life Technologies. Processing of the Ion Torrent Personal Genome Machine (PGM) runs was achieved with the Torrent Suite version 4.4.3. The Coverage Analysis plugin was used to calculate coverage efficiency and read depth. The Ion Reporter v4.6 was used to identify and annotate variants.

### DNA sequencing using the sanger method

Pathogenic and novel variants were confirmed by custom oligonucleotide primers designed to flank the variant of interest in order to prepare PCR fragments for sequencing. PCR was performed at optimal condition for each primer pair and products were purified using ethanol precipitation. DNA sequencing was performed using the BigDye v. 3.1 and analyzed on the DNA Analyzer 3500 from Life Technologies. Additionally, TaqMan® SNP genotyping assays, either custom-designed or pre-made, were utilized in the validation process or screening further cases.

## Results

We have adopted the amplicon-based targeted sequencing approach in order to develop a more efficient method for the detection of mutations affecting the deafness genes. In this approach, sequencing is limited to the genes known or has been previously demonstrated to play a role in causing HI. (Table 1) lists the genes included in this panel obtained from the deafness variation database (http://deafnessvariationdatabase.org).

**Table 1:** Genes included in our HI panel

Gene	Coverage (%)	Gene	Coverage (%)	Gene	Coverage (%)	Gene	Coverage (%)
ACTG1	56.62	GIPC3	98.9	OTOA	97.82	TSPEAR	97.54
AIFM1	100	GJB2	100	OTOF	95.41	USH1C	97.21
ALMS1	99.25	GJB3	78.86	OTOG	98.3	USH1G	100
ATP2B2	100	GJB6	100	OTOGL	99.71	USH2A	98.87
CABP2	100	GPR98	99.42	P2RX2	80.21	WFS1	97.63
CACNA1D	100	GPSM2	100	PCDH15	98.27	WHRN	100
CATSPER2	100	GRHL2	100	PNPT1	97.07	MIR-182	100
CCDC50	100	GRXCR1	100	POU3F4	100	MIR-183	66
CDH23	99.55	HGF	99.4	POU4F3	100	MIR96	100
CEACAM16	94.39	ILDR1	95.7	PRPS1	100	DFNB59	100
CIB2	95.87	KCNQ1	86.04	PTPRQ	97.5	ESRRB	91.35
CLDN14	88.36	KCNQ4	87.25	RDX	100	TMPRSS3	100
CLPP	99.78	LARS2	98.63	SERPINB6	97.18		
CLRN1	100	LHFPL5	100	SIX1	95.77		
COCH	100	LOXHD1	99.72	SLC26A4	100		
COL11A2	99.9	LRTOMT	100	SLC26A5	100		
CRYL1	99.04	MARVELD2	100	SMAD4	100		
CRYM	100	MSRB3	100	SMPX	100		
DFNA5	100	MYH14	97.53	STRC	99.82		
DIABLO	100	MYH9	98.11	TECTA	98.28		
DIAPH1	98.49	MYO15	94.22	TJP2	98.17		
DSPP	80.44	MYO1A	100	TMC1	96.54		
ESPN	73.28	MYO3A	100	TMIE	79.84		
EYA1	100	MYO6	100	TPRN	93.57		
EYA4	100	MYO7A	98.72	TRIOBP	92.17		

Eighty-seven genes were selected and custom primers were designed and manufactured through the ampliseq portal (http://ampliseq.com). The design resulted in an excellent coverage of 97.42 % generating 2697 amplicons with a size range of 125–275 bp in two pools and generating 500.44 kb of DNA sequence. Identified variants were confirmed by Sanger sequencing.

**Fig. 1:**
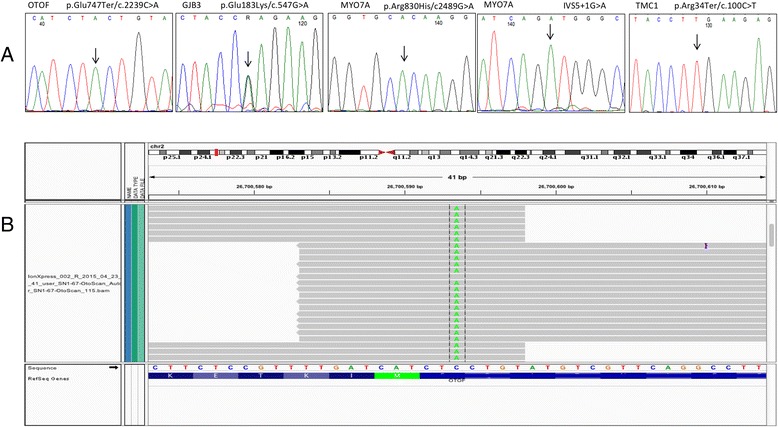
Sanger sequencing validation of the identified pathogenic variants (**a**) and the Integrated Genome Viewer view of the Ion Torrent BAM file generated by the targeted sequencing using our custom-made HI panel demonstrating the detection of the p. Glu747Ter/c.2239C > A mutation in the OTOF gene (**b**)

We have managed to screen 25 individuals affected with HI using this approach. Additionally, we have screened samples from 6 healthy volunteers with no history of HI as a control. Seven patients were found to have pathogenic (according to clinvar database) mutations affecting OTOF, MYO7A, TMC1 and GJB3 genes (Table 2; Fig. 1). The homozygous c.2239C > A change in the OTOF gene causes a premature termination of the protein at p. Glu747Ter. This mutation was identified in a brother and sister suffering from severe to profound HI. The homozygous IVS5 + 1G > A change in the MYO7 gene is a mutation affecting the 5′ splice site and was found in a brother and sister with severe to profound HI in our screen. The p. Arg830His in MYO7A gene is a very rare SNP (single nucleotide polymorphisms) with a reported minor allele frequency of 0.0001556 and classified as likely pathogenic by the ClinVar database (http://www.ncbi.nlm.nih.gov/clinvar/). This variant was identified in two of our patients with severe to profound HI, albeit one of the patient harbored the mutation in a heterozygous state thus could not sufficiently account for the disease. The c.100C > T, p. Arg34Ter variant in the TMC1 gene is a pathological change according to ClinVar database. The c.100C > T, p. Arg34Ter homozygous change in the TMC1 gene causes the premature termination of the protein and found only in one patient. The rs74315318 SNP is c.547G > A change causing a p. Glu183Lys alteration in the GJB3 gene. This variant is reported to be pathological in an autosomal dominant fashion in the ClinVar database.

**Table 2:** Pathogenic mutations identified in our cohort

Position	genotype	gene	transcript	coding	protein	diagnosis
chr2:26700593	A/A	OTOF	NM_194248	c.2239G > T	p. Glu747Ter	Severe to profound
chr1:35250910	G/A	GJB3	NM_001005752	c.547G > A	p. Glu183Lys	Severe to profound
chr11:76890902	A/A	MYO7A	NM_000260	c.2489G > A	p. Arg830His	Severe to profound
chr11:76867138	A/A	MYO7A	NM_000260	c.470 + 1G > A	IVS5 + 1G > A	Severe to profound
chr9:75309494	T/T	TMC1	NM_138691	c.100C > T	p. Arg34Ter	Severe to profound

Although we could not identify additional known pathogenic mutations in our cohort, we have identified 7 patients with a possible genetic aberration underlying their HI. In these patients, two novel or rare SNVs were identified affecting the same gene (Table 3). In particular, in the OTOF and PCDH15 genes. The rare damaging SNPs [minor allele frequency (MAF) of 0.001 and 0.006, respectively] identified in combination in the PCDH15 gene were recurrent in 3 unrelated patients. Furthermore, we have designed custom-made TaqMan® SNP genotyping assays targeting such mutations in order to test whether the variants identified in this screen recur in other unrelated patients. Interestingly, the combination identified by the panel was not found in 80 additional HI patients or non-HI controls.

**Table 3:** Novel or rare variants identified in our screen

Case	Position	ref	genotype	MAF	gene	protein	coding	SIFT (score)	polyphen	dbsnp	transcript
147	chr2:26689695	G	G/C	NA	OTOF	p. Pro1463Ala	c.4387C > G	Damaging (0)	Probably damaging (0.965)	Novel	NM_194248.2
	chr2:26750782	G	G/A	0.008	OTOF	p. Arg49Trp	c.145C > T	Damaging (0)	Probably damaging (1)	rs61746568	
144	chr2:26695500	A	A/C	0.009	OTOF	p. Cys1251Gly	c.3751 T > G	Tolerated (0.46)	Benign (0)	rs41288773	NM_194248.2
	chr2:26750782	G	G/A	0.008	OTOF	p. Arg49Trp	c.145C > T	Damaging (0)	Probably damaging (1)	rs61746568	
151	chr2:26696374	C	C/T	0.006	OTOF	p. Arg1157Gln	c.3470G > A	Tolerated (1)	Probably damaging (1)	rs56054534	NM_194248.2
	chr2:26781384	C	C/T	0	OTOF	p. Arg19Gln	c.56G > A	Damaging (0.006)	Probably damaging (1)	rs200316189	
197	chr10:55779975	C	C/A	0.001	PCDH15	p. Ala915Ser	c.2743G > T	Damaging (0.05)	Probably damaging (1)	rs139175351	NM_001142763.1
	chr10:55782743	A	A/G	0.006	PCDH15	p. Ile817Thr	c.2450 T > C	Damaging (0.023)	Probably damaging (0.998)	rs61731363	
350	chr10:55779975	C	C/A	0.001	PCDH15	p. Ala915Ser	c.2743G > T	Damaging (0.05)	Probably damaging (1)	rs139175351	NM_001142763.1
	chr10:55782743	A	A/G	0.006	PCDH15	p. Ile817Thr	c.2450 T > C	Damaging (0.023)	Probably damaging (0.998)	rs61731363	
888	chr10:55779975	C	C/A	0.001	PCDH15	p. Ala915Ser	c.2743G > T	Damaging (0.05)	Probably damaging (1)	rs139175351	NM_001142763.1
	chr10:55782743	A	A/G	0.006	PCDH15	p. Ile817Thr	c.2450 T > C	Damaging (0.023)	Probably damaging (0.998)	rs61731363	
873	chr3:121712010	A	A/G	NA	ILDR1	p. Val529Ala	c.1586 T > C	Tolerated (1)	Benign (0.39)	Novel	NM_001199799.1
	chr7:129414567	C	C/T	NA	MIR96					rs370173345	NR_029512.1

## Discussion and conclusions

We have screened 25 HI patients using a custom-made targeted sequencing panel to simultaneously screen 87 genes known to play a role in HI. Known pathogenic mutations were identified in 7 patients which gives a diagnostic rate of 28 %. The rs74315318 SNP is a c.547G > A change causing a p. Glu183Lys alteration in the GJB3 protein. This variant is reported to be pathological in the ClinVar database [14] according to a published report of its identification in Chinese families with autosomal dominant hearing loss [15]. We have also identified a homozygous c.2239C > A change in the OTOF gene causing premature termination of protein at p. Glu747Ter. This mutation was identified in a brother and sister suffering from severe to profound HI. Although not reported in the dbSNP, this variant was previously identified in a Libyan family [16]. The c.100C > T, p. Arg34Ter homozygous change in the TMC1 gene causes the premature termination of the protein and has been previously reported in more than 23 probands with sensorineural hearing loss [17–19]. We have identified this variant in the homozygous state in one patient with profound HI. The homozygous IVS5 + 1G > A change in the MYO7A gene is a mutation affecting the 5′ splice site and was found in a brother and sister with severe to profound HI in our screen. This mutation was reported only once previously in a Tunisian family enrolled in a study of Usher 1B syndrome [20]. The p. Arg830His in the MYO7A gene is a very rare SNP with a minor allele frequency of 0.0001556 as reported by the exome aggregation consortium (ExAC) data set [21]. This variant was identified in two of our patients with profound HI.

Genetic factors underlying HI patients from Saudi Arabia are not fully elucidated and therefore, other mutations may play a role. For example, we have identified in this study a C > T change in the sequence of the MIR-96 microRNA which has been recently shown to play a role in hearing [22]. This SNP is extremely rare and according to the ExAC browser [21] carries a minor allele frequency of 0.0001428. This mutation is also conserved and lies close to the +57 T > C mutation recently shown to cause autosomal dominant hearing loss in Italian families by altering pre-miRNA processing [23]. This variant was identified in a patient with severe to profound hearing loss. In addition, we have identified a patient that harbored a rare rs369424114 variant in the MYO7A gene. This variant has a minor allele frequency of 0.0006688 and found predominantly in the south Asian population [21].

An interesting observation in this cohort is the co-existence of two damaging variants of PCDH15 gene in 3 unrelated patients. The reported MAF for the p. Ala915Ser and the p. Ile817Thr variants is 0.001 and 0.006, respectively. Additionally, a novel OTOF gene variant, p. Pro1463Ala, co-existed with another rare OTOF variant, p. Arg49Trp, (MAF = 0.008) in one patient. Another patient harbored the latter variant with the rare p. Cys1251Gly variant (MAF = 0.009) affecting the same gene. A third unrelated patient harbored the rare p. Arg1157Gln variant in the OTOF gene (MAF = 0.001) as well as another rare variant, p. Arg19Gln, affecting the same gene. The co-existence of such rare or novel variants may reflect a new state of pathogenicity of single nucleotide variants affecting the OTOF and PCDH15 genes in our cohort.

In conclusion, our study shows that targeted sequencing approach is a very useful strategy for the identification of mutations affecting the HI genes. However, further work is necessary to identify and characterize novel variants in each ethnic group. Targeted sequencing is relatively fast and cost effective compared to whole-exome sequencing which renders possible the screening of large cohorts of sporadic HI patients. Additionally, targeted sequencing combined with next generation sequencing can be utilized as an aid for genetic counseling of families affected with hearing impairment. Furthermore, such test can be applied as a premarital screening tool at a national level in order to significantly reduce the burden of HI on the society.

## Abbreviations

ExAC: Exome aggregation consortium
HI: Hearing impairment
MAF: Minor allele frequency
MPS: Massively parallel sequencing
NSHL: Non-syndromic hearing loss
PGM: Personal genome machine
SNPs: Single nucleotide polymorphisms
WES: Whole-exome sequencing
